# A high throughput screen for TMPRSS2 expression identifies FDA-approved and clinically advanced compounds that can limit SARS-CoV-2 entry

**DOI:** 10.21203/rs.3.rs-48659/v1

**Published:** 2020-08-14

**Authors:** Yanwen Chen, Travis B. Lear, John W. Evankovich, Mads B. Larsen, Bo Lin, Irene Alfaras, Jason R. Kennerdell, Laura Salminen, Daniel P. Camarco, Karina C. Lockwood, Jie Liu, Michael M. Myerburg, John F. McDyer, Yuan Liu, Toren Finkel, Bill B. Chen

**Affiliations:** 1 Aging Institute, University of Pittsburgh/UPMC, Pittsburgh, PA 15219, USA; 2 Department of Gastroenterology, Ruijin Hospital, Shanghai Jiaotong University School of Medicine, Shanghai 200025, China; 3 Department of Medicine, Acute Lung Injury Center of Excellence, University of Pittsburgh, Pittsburgh, PA 15213, USA; 4 Vascular Medicine Institute, University of Pittsburgh, Pittsburgh, PA, 15213, USA; 5 McGowan Institute for Regenerative Medicine, University of Pittsburgh, Pittsburgh, PA 15219, USA; 6 Department of Medicine, Division of Cardiology, University of Pittsburgh, Pittsburgh, PA 15213, USA

## Abstract

SARS-CoV-2 (2019-nCoV) is the pathogenic coronavirus responsible for the global pandemic of COVID-19 disease. The Spike (S) protein of SARS-CoV-2 attaches to host lung epithelial cells through the cell surface receptor ACE2, a process dependent on host proteases including TMPRSS2. Here, we identified small molecules that can reduce surface expression of TMPRSS2 using a 2,700 FDA-approved or current clinical trial compounds. Among these, homoharringtonine and halofuginone appear the most potent agents, reducing endogenous TMPRSS2 expression at sub-micromolar concentrations. These effects appear to be mediated by a drug-induced alteration in TMPRSS2 protein stability. We further demonstrate that halofuginone modulates TMPRSS2 levels through proteasomal-mediated degradation that involves the E3 ubiquitin ligase component DDB1- and CUL4-associated factor 1 (DCAF1). Finally, cells exposed to homoharringtonine and halofuginone, at concentrations of drug known to be achievable in human plasma, demonstrated marked resistance to SARS-CoV-2 pseudoviral infection. Given the safety and pharmacokinetic data already available for the compounds identified in our screen, these results should help expedite the rational design of human clinical trials designed to combat COVID-19 infection.

## Introduction

SARS-CoV-2 is a coronavirus first described in Wuhan, China that shares many similarities with other pathogenic beta coronaviruses, including the SARS-CoV virus and the MERS coronavirus (1). After emergence of SARS-CoV, several groups identified the molecular and cellular pathways through which this virus attaches, enters, and replicates in host respiratory epithelial cells (2–4). To mediate cell entry, the SARS-CoV viral Spike (S) glycoprotein is recognized by the extracellular receptor angiotensin converting enzyme 2 (ACE2) on host respiratory epithelial cells (5, 6). A second critical process, S protein priming, is essential to complete viral entry and spread (7). S protein priming is a process wherein the spike protein is cleaved by host proteases. Two separate classes of proteases were shown to prime SARS-CoV S protein - endo-lysosomal proteases in the cathepsin family, and the plasma membrane associated protease, TMPRSS2 (3, 8). The viral entry mechanisms elucidated for SARS-CoV infection appear to be shared by SARSCoV-2 (9, 10). Additionally, SARS-CoV-2 uniquely possesses a “furin-like” cleavage site and is subject to processing during viral packaging by the intracellular proprotein convertase furin (11–14).

Host-directed efforts to limit SARS-CoV-2 infection could logically entail strategies to manipulate either ACE2 or the S protein priming step. However, in animal models, reducing ACE2 activity appears to increase lung injury in response to SARS-CoV infection or sepsis (6, 15). In contrast, mice lacking TMPRSS2 exhibit no discernable basal phenotype (16), but demonstrate protection from acute SARS-CoV infection (3). These observations led us, and others (9, 17), to hypothesize that reducing or inhibiting TMPRSS2 may be an attractive strategy to mitigate SARS-CoV-2 pathogenicity. Given SARS-CoV-2’s rapid spread, coupled with the high burden of acute respiratory failure and death, there is an urgent need for therapies. We believe the most rapid and effective near-term approach is to identify current FDA approved drugs that can be re-purposed to block viral entry and or spread. As such, we have executed a screen of approximately 2,700 FDA-approved or current clinical trial compounds to ascertain their ability to be repurposed to reduce TMPRSS2 abundance and prevent SARS-CoV-2 entry in lung epithelial cells. Additionally, we provide insight into the cellular regulation of TMPRSS2 – notably that it is targeted for E3 ligase driven ubiquitination and proteasomal degradation.

## Result

**Figure 1:**
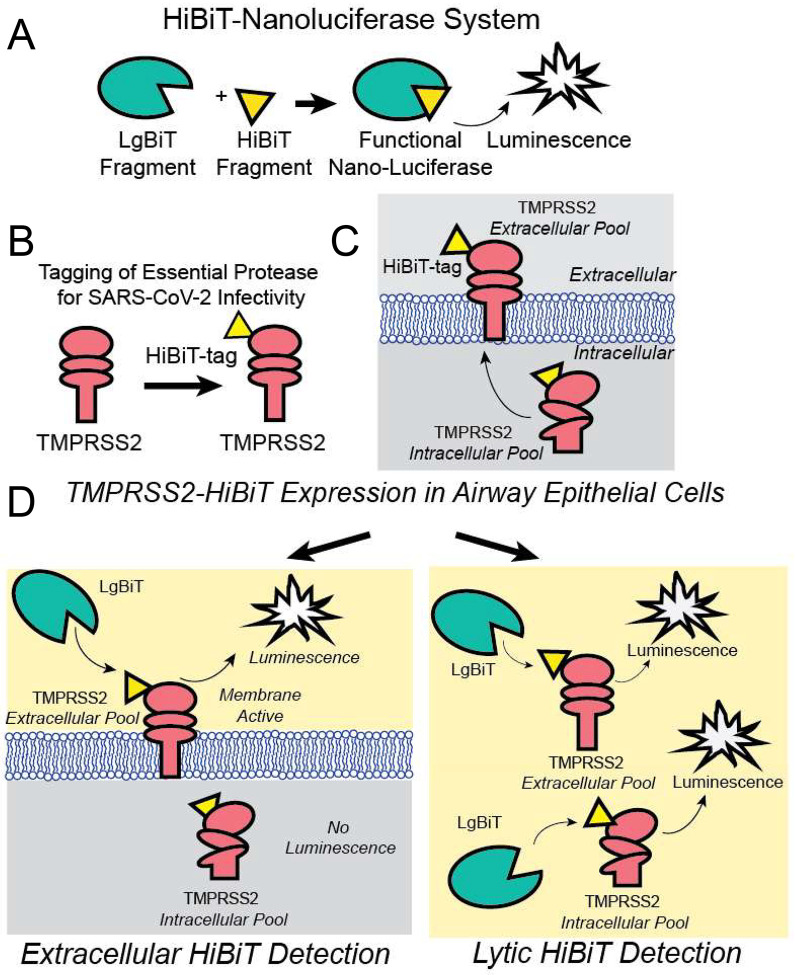
Schematic of TMPRSS2-HiBiT detection for high throughput screen. (**A**) The split nano-luciferase components LgBiT and HiBiT can interact to form a functional enzyme that generates luminescence. (**B**) Human TMPRSS2 cDNA was C-terminally tagged with a HiBiT sequence on a domain that is extracellular when TMPRSS2 is present in the plasma membrane. (**C**) The TMPRSS2-HiBiT construct was expressed in human airway cells where it exists in an intracellular pool and a plasma membrane-associated pool. (**D**) Non-lytic extracellular HiBiT detection results in LgBiT-HiBiT complementation solely with the pool of plasma membrane localized TMPRSS2. Following extracellular HiBiT detection, cells are lysed and the total TMPRSS2- HiBiT is then quantified.

**Figure 2:**
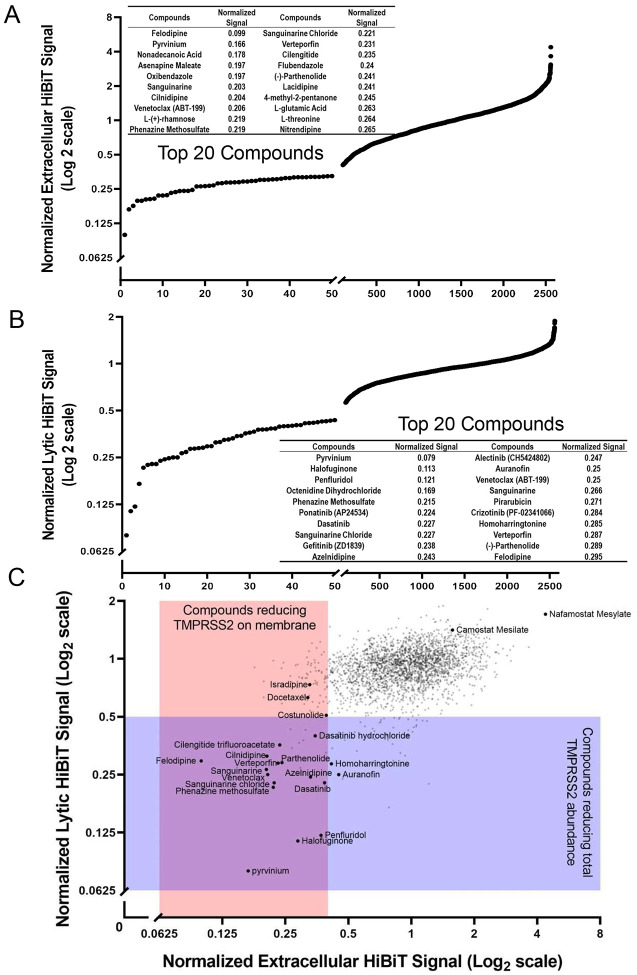
Screening of airway cells with a FDA-approved or clinically active compound library for agents that reduce TMPRSS2-HiBiT levels. (**A**) HTS results from the non-lytic extracellular HiBiT detection of TMPRSS2-HiBiT. The top 20 compounds are specified. (**B**) Lytic HiBiT detection reflecting total cellular TMPRSS2-HiBiT; top 20 compound hits are listed. (**C**) Scatterplot of hit compounds from both screens. Compounds that reduce membrane TMPRSS2-HiBiT signal (non-lytic extracellular HiBiT detection) are shown in pink, compounds that reduce total TMPRSS2-HiBiT signal (lytic HiBiT detection) are shown in purple. Some individual compounds are specified.

In order to assess agents that might alter TMPRSS2 expression, we first engineered full length human TMPRSS2 to express a C-terminal 11 amino acid tag (HiBiT), which produces a bioluminescent signal when combined with a complementary protein (LgBiT) and a furimazine substrate (18). The HiBiT-tagged TMPRSS2 construct was expressed in human bronchial epithelial cells (Beas-2B) allowing us to detect either plasma membrane associated TMPRSS2, using bioluminescence associated with a non-lytic nano-luciferase (Nano-Luc) reagent or total TMPRSS2 expression, using a lytic luciferase agent (Fig. 1). Elements of the screen were optimized and miniaturized to a 384-well format. We calculated the Z’ score to be 0.38, and thus suitable for high-throughput applications (fig. S1). We next screened a chemical library (FDA-approved and current clinical trial drugs) consisting of ~2,700 compounds for their capacity to decrease TMPRSS2-dependent Nano-Luc activity. We assayed the compounds effect on both the extracellular plasma membrane associated and total TMPRSS2-HiBiT signal using the non-lytic and lytic luciferase reagent. At a compound concentration of 10 μM, this screen identified ~100 well-characterized drugs that produced a >50% reduction in either extracellular or total TMPRSS2-HiBiT signal (Fig. 2). A number of compounds were positive in both screens (Fig. 2c: intersection of purple and pink rectangles). In contrast, the agent nafamostat, a serine protease inhibitor, resulted in the opposite effect, as treatment with this agent potently increased TMPRSS2 expression (Fig. 2c). This compound is known to block TMPRSS2 enzymatic activity and thereby serve as a potential strategy to limit SARS-CoV and MERS acute infection of cells (19). A related compound, camostat, is also known to enzymatically inhibit TMPRSS2, and was recently shown to block acute SARS-CoV-2 infection (9), prompting the initiation of a clinical trial for this agent in COVID-19 patients (ClinicalTrials.gov Identifier: NCT04321096). However, our results suggest there might be a feedback mechanism through which TMPRSS2 enzymatic activity regulates overall levels of the protease. As such, these observations serve as a potentially cautionary note for using TMPRSS2 enzymatic inhibitors as a means to reduce coronavirus infections (9, 19).

**Figure 3:**
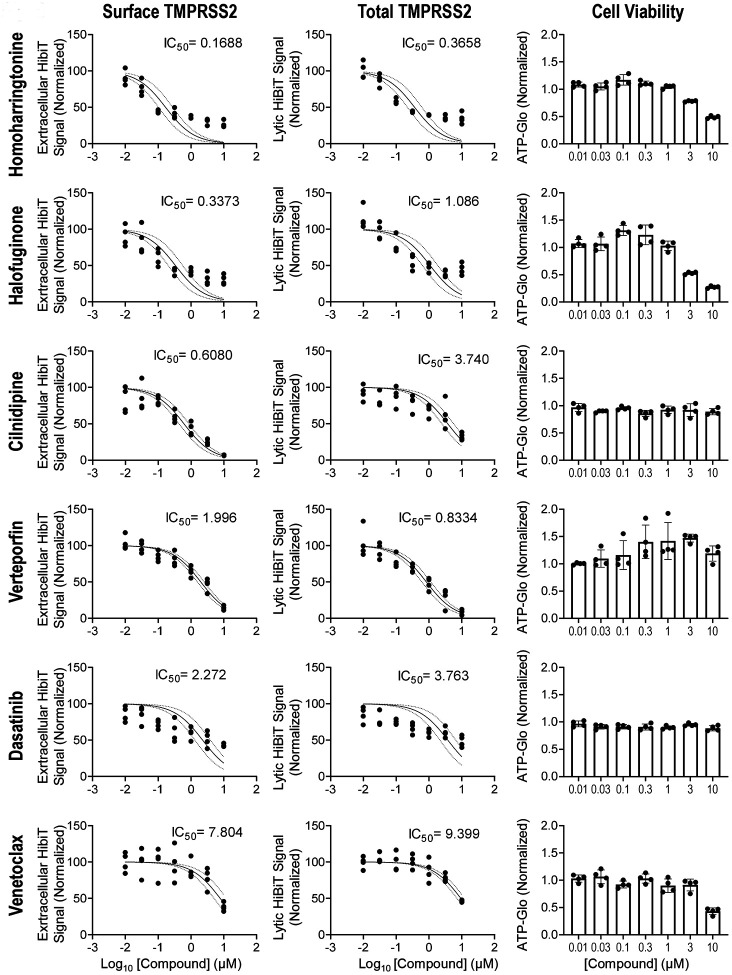
Determination of the potency for a subset of identified compounds. A selection of six of the most promising drugs were assessed for their activity (IC50) to inhibit TMPRSS2 expression extracellularly (first column) or to inhibit total TMPRSS2 (middle column). An assessment of cellular toxicity for each compound (CellTiter-Glo) is shown in the last column. The remaining activity profiles for other identified agents are found in the supplementary figures. data are mean +/− SEM (n=4).

The most potent compounds (24 in total) were subsequently cherry-picked and tested extensively in dose-course studies for their effect on the extracellular and intracellular TMPRSS2-HiBiT signal in airway epithelial cells (Fig. 3 and fig. S2). These agents were also directly assessed by Western blot analysis for TMPRSS2-HiBiT protein expression (fig. S3). An assessment of the compound’s toxicity, as measured by the drug’s effect on cell number (using CellTiter-Glo 2.0) was also determined, so as to give an estimate of each compounds’ potential therapeutic index (Fig. 3 and fig. S2). As noted, many agents, including homoharringtonine (approved for chronic myeloid leukemia), halofuginone (in clinical trials for scleroderma) and cilnidipine (a calcium-channel blocker, antihypertensive agent approved in Asia and in some European countries), were effective in reducing extracellular TMPRSS2 expression at sub-micromolar concentrations. In general, these agents were also effective in reducing total TMPRSS2 as well, although for certain agents (e.g. cilnidipine) this required a substantially higher concentration of drug.

**Figure 4:**
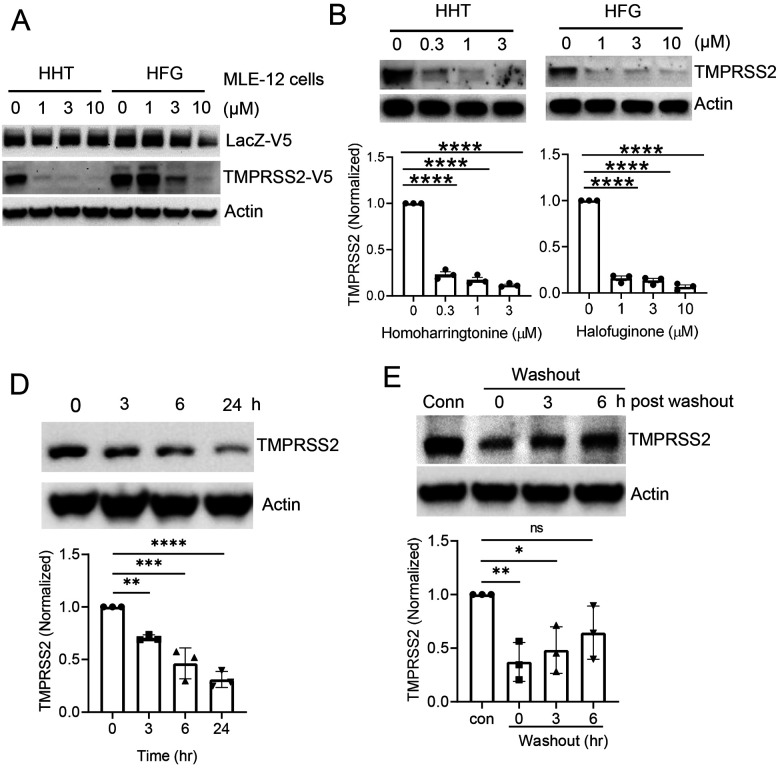
Homoharringtonine and halofuginone potently reduce TMPRSS2 protein levels. (**A**) Immunoblot data from MLE-12 cells co-expressing LacZ-V5 and TMPRSS2-V5 treated with homoharringtonine (HHT) or halofuginone (HFG) at the indicated concentrations for 18 hr. (**B-C**) Immunoblot analysis of endogenous TMPRSS2 protein level in Caco-2 cells treated for 18 hr with HHT (**B**) or HFG (**C**). TMPRSS2 densitometry is shown, data are mean +/− SEM (n=3). (**D**) Time- course treatment of HFG-treated Caco-2 cells (3 μM). TMPRSS2 densitometry is shown, data are mean +/− SEM (n=3). (**E**) Immunoblot analysis of Caco-2 cells treated with HFG for 18hr prior to removing the drug, adding fresh media, and then analyzing the protein recovery time course. (All TMPRSS2 densitometry that is shown represents mean +/− SEM (n=3). Actin is shown as a loading control. NS, P>0.05; *, P <0.05; **, P < 0.01; ***, P <0.001; ****, P<0.0001 relative to 0 time point or control, or as indicated by one-way ANOVA with Dunnett’s test of multiple comparisons (B-E).

The clinical utility of any agent identified ultimately depends on the ability of the potential therapeutic compound to reach presumed therapeutic levels in patients. As such, we compared the measured IC50 from our screen to the known pharmacokinetic properties of the identified agents. While we would ideally have preferred knowing the concentration of each drug in the presumptive target organ (e.g. lung), such information is not currently publicly available for many of these agents. As such, as an approximation, we leveraged the known concentration of each of these compounds in human plasma. From this exercise, homoharringtonine and halofuginone emerged as the candidates most likely to be clinically effective. We next sought to confirm that the reduction in the Nano-Luciferase signal observed in our screen by these two agents was not cell-type specific, dependent on the HiBiT tag, or a transcriptional effect of these compounds on the heterologous CMV promoter used to drive expression of our HiBiT-tagged TMPRSS2 construct. Using the same CMV promoter, we transiently expressed either a V5-tagged TMPRSS2 or a V5-tagged LacZ protein in the mouse respiratory epithelial cell line MLE-12. As noted in Figure 4a, both agents seem to selectively reduce TMPRSS2 expression in cells.

We then asked whether these compounds were effective in reducing endogenous TMPRSS2 expression. We chose the intestinal epithelial cell line Caco-2, since these cells are known to express high levels of TMPRSS2 and to be permissive for both SARS-CoV and SARS-CoV-2 infection (9, 20). Moreover, evidence suggests that the human intestinal tract may be an alternative entry point for coronaviruses (21). Treatment of Caco-2 cells for 18 hours with either homoharringtonine or halofuginone resulted in a marked reduction in endogenous TMPRSS2 protein expression (Fig. 4b, c). As expected, the observed decrease in TMPRSS2 protein expression was not a consequence of a drug-induced reduction in TMPRSS2 transcription, which if anything, modestly increased with compound treatment (fig. S4). We also noted qualitatively similar decreases in TMPRSS2 protein levels in homoharringtonine or halofuginone-treated Calu-3 cells, a human lung cancer cell line that is also permissive for SARS-CoV-2 infection (9) (fig. S5a, b). A time course demonstrated that the effects of drug treatment were relatively rapid, with a substantial reduction in TMPRSS2 expression evident within three hours (Fig. 4d, Fig. S5c). Similarly, levels of TMPRSS2 returned close to baseline roughly six hours after drug removal (Fig. 4e, Fig. S5d).

**Figure 5:**
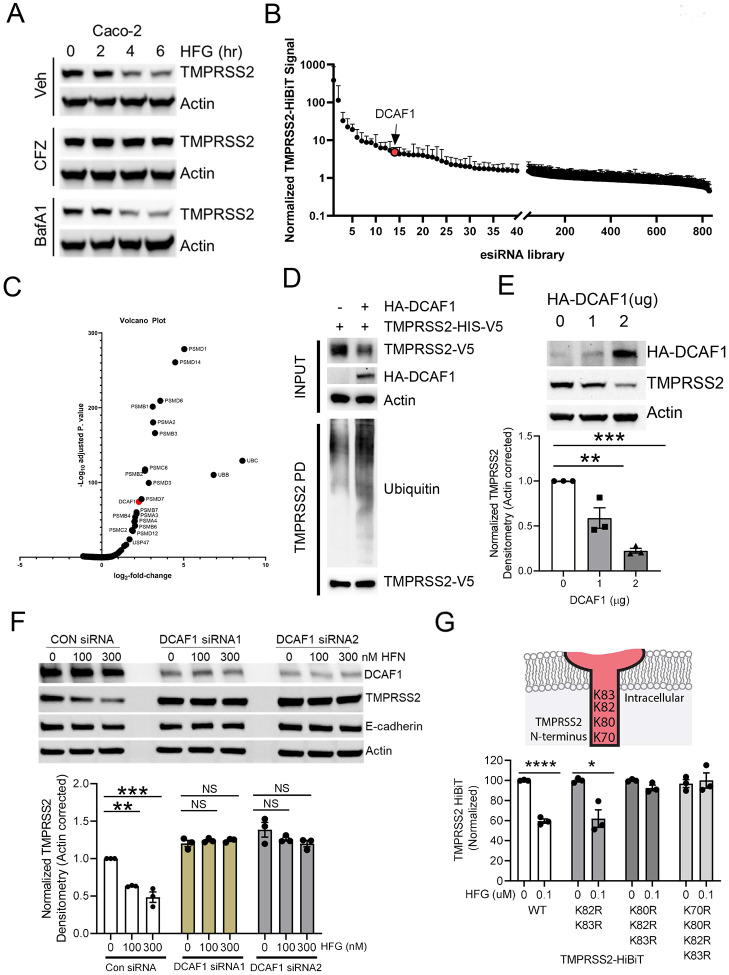
TMPRSS2 is degraded through ubiquitin proteasome system, which is required for HFG efficacy. (**A**) Immunoblotting assay of Caco-2 cells treated with HFG in combination with carfilzomib (CFZ) or bafilomycin A1 (BafA1) and probed for TMPRSS2. (**B-C**) TMPRSS2-HiBiT signal was measured following siRNA knockdown of ubiquitination-related machinery. **(B)** Ubiquitination siRNA library screening results ordered by increase in TMPRSS2-HiBiT signal. The E3 ligase DCAF1 was detected as a top hit. (**C**). Volcano plot of TMPRSS2-HiBiT signal screening with Ubiquitination siRNA library from n=3 independent screening assays. Statistical significance is plotted against log2-fold change in TMPRSS2-HiBiT signal. Top hits are annotated. **(D)** DCAF1 co-expression increases TMPRSS2 ubiquitination. **(E)** Immunoblot analysis of Beas-2B cells with increasing expression of DCAF1, TMPRSS2 densitometry shown represents mean +/− SEM (n=3). **(F)** Immunoblot analysis of Beas-2B cells with control or DCAF1 siRNA treatment followed by HFG treatment. TMPRSS2 densitometry represent mean −/+ SEM (n=3). **(G)** TMPRSS2 intracellular lysines were assayed for their responsiveness to HFG, data represent mean +/− SEM (n=3). Actin is shown as a loading control throughout, and E-cadherin is added as an additional plasma membrane loading control. NS, P > 0.05; *, P < 0.05; **, P < 0.01 ***, P < 0.001; ****, P<0.0001 relative to control, or as indicated by one-way ANOVA with Dunnett’s test of multiple comparisons (E-F), or two-way unpaired t-test (G).

While homoharringtonine is a relatively toxic agent with an adverse side effect profile (22, 23), halofuginone, has a very mild toxicity profile and is generally well tolerated (24, 25). Given the potential safety advantages of halofuginone, we therefore sought to gain additional understanding of how this agent modifies TMPRSS2 expression. Given the rapid decay of TMPRSS2 upon halofuginone treatment, we initially focused our efforts on determining the means of protein degradation. While the lysosomal inhibitor Bafilomycin A1 was without effect, the effects of halofuginone on TMPRSS2 protein levels were largely abrogated in the presence of the proteasomal inhibitor carfilzomib (Fig 5a). This argues that TMPRSS2 likely undergoes clearance through the ubiquitin-proteasomal system (UPS). To gain further mechanistic insight into this process, we employed Beas-2B cells stably expressing TMPRSS2-Hibit to perform a high-throughput screen using siRNAs targeting ~800 components of the UPS including siRNAs targeting ubiquitin, proteasome subunits, E1, E2, E3, deubiquitinases (DUBs), and E3 ligases. Knockdown of ubiquitin or proteasome subunits increased TMPRSS2 HiBiT signal robustly, consistent with our assumption that the protein is subject to UPS-mediated turnover (Fig 5b–c). We also observed that knockdown of the E3 ligase subunit DCAF1 increased TMPRSS2 abundance (red dots) (Fig. 5b–c). Overexpression of DCAF1 decreased TMPRSS2-V5 protein in total cell lysate, and also increased poly-ubiquitination of TMPRSS2, suggesting a role of this E3 component in TMPRSS2 ubiquitination and half-life (Fig. 5d). Overexpression of DCAF1 also dose-dependently decreased endogenous TMPRSS2 (Fig. 5e). To further establish the mechanism by which halofuginone modulates TMPRSS2 stability, we performed siRNA-mediated knockdown of DCAF1 in the setting of halofuginone treatment. In control knockdown cells, halofuginone reduced TMPRSS2 expression without altering the levels of another cell surface protein, E-cadherin. In cells subjected to DCAF1 siRNA mediated knockdown (using two separate siRNAs), we noted an increase in basal levels of TMPRSS2. Of note, knockdown of DCAF1 knockdown abrogated the effects of halofuginone on TMPRSS2 protein levels (Fig. 5f). We next took advantage of the observation that lysine residues in target proteins are often the site of E3-mediated ubiquitination (26). TMPRSS2 contains four lysine residues on its intracellular domain, with three lysines closely clustered (K80, K82 and K83). In contrast to the wild-type protein, or to a site directed mutant in which only two lysines were altered, a site-directed mutant of TMPRSS2 in which all three lysines were converted to arginine was resistant to the effects of halofuginone (Fig. 5g). This suggests that DCAF1 is a potential E3 ligase subunit that regulates TMPRSS2 stability through ubiquitination. Of note, halofuginone has also been postulated to act as a glutamyl-prolyl-tRNA synthetase inhibitor, inhibiting translation of a subset of proteins (27). While halofuginone does not exhibit strict substrate specificity, it has been proposed that halofuginone reduces the rate of translation of “proline-rich” proteins. In that regard, we note that TMPRSS2 sequence contains 7% proline residues. Indeed, we observed that halofuginone can inhibit the *in vitro* and *in vivo* synthesis of TMPRSS2 in a proline-dependent fashion (fig. S6a, b). As such, it seems likely that halofuginone can affect both the synthesis and degradation of TMPRSS2.

**Figure 6:**
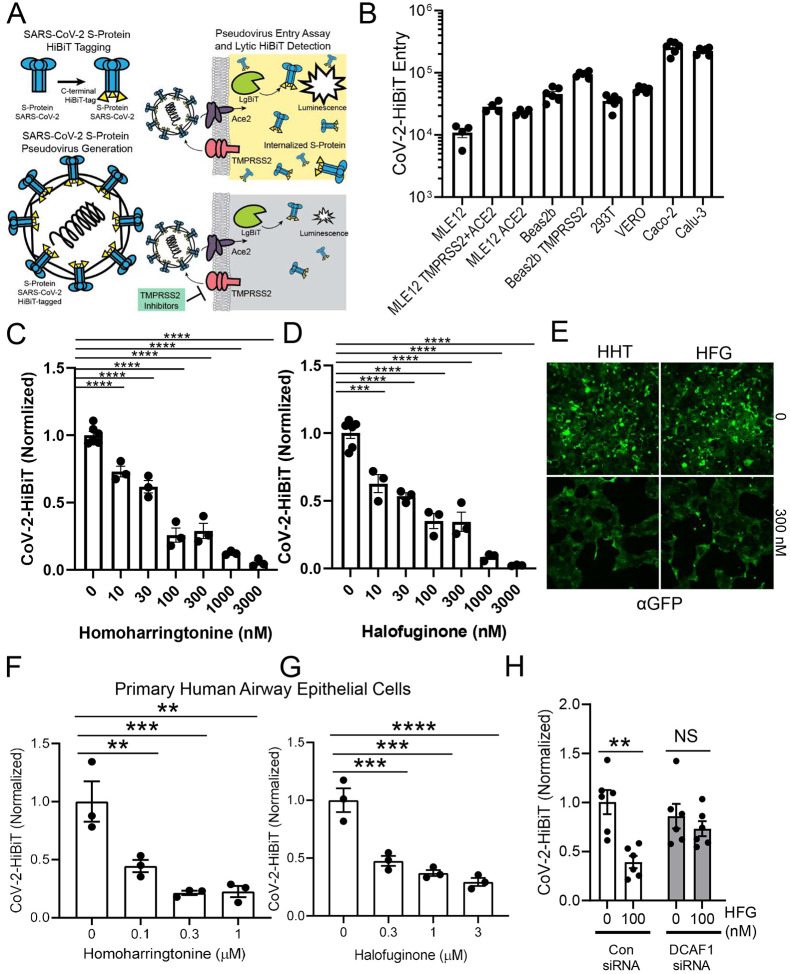
Agents that reduce TMPRSS2 expression markedly inhibit SARS-CoV-2 pseudoviral infection. (**A**) Schematic of pseudoviral construction and assay. The S protein of SARS-CoV-2 was C-terminally tagged with HiBiT. (**B**) Level of viral transduction in various cell lines plotted on a logarithmic scale. Calu-3 and Caco-2 cells had the highest observed rates of infection. (**C-D**) Effects of increasing concentrations of homoharringtonine (HHT) (**C**) or halofuginone (HFG) (**D**) on SARS-CoV-2 pseudoviral infection. (**E**) Effects of HHT or HFG on pseudoviral-mediated GFP expression, determined by immunostaining. (**F-G**) SARS-CoV-2 pseudoviral infection of primary human bronchial epithelial cells in the presence of increasing concentrations of HHT (**F**) and HFG (**G**). (**H**) SARS-CoV-2 pseudoviral infection of Caco-2 cells transfected with DCAF1 siRNA along with HFG treatment (100nM). All SARS-CoV-2 pseudoviral data is corrected to cell number as determined by CellTiter-Glo. **, P<0.01; ***, P <0.001; ****, P<0.0001 relative to 0 time point or control, or as indicated by one-way ANOVA with Dunnett’s test of multiple comparisons (F-G), or a two-way ANOVA with Tukey’s test of multiple comparisons (H)

We next asked whether the agents we have identified could effectively limit viral infection. Given the biosafety issues of working with live SARS-CoV-2, we addressed this scientific question by creating a pseudovirus, which incorporated the SARS-CoV-2 Spike protein. For the sake of viral entry, this pseudovirus faithfully recapitulates COVID-19 infection. Of note, this strategy has been employed recently by other groups (9, 28, 29). In our case, we also molecularly tagged the Spike protein with the HiBiT sequence to allow rapid and sensitive detection of viral infection. The strategy employed is diagramed in Figure 6a. We first assessed the ability of our SARS-CoV-2 pseudovirus to infect various cell lines, and as previously noted, both Caco-2 and Calu-3 cells appeared to be highly permissive (Fig 6b), consistent with the known high level expression of both ACE2 and TMPRSS2 in these cell lines (9, 20, 30). We then asked whether homoharringtonine or halofuginone were biologically active in reducing SARS-CoV-2 infection. Remarkably, both agents were able to affect a 50% reduction in the level of viral infection at concentration ~30 nM (Fig. 6c, d). Similar, albeit slightly less potent effects, were also seen in Calu-3 cells (fig. S7a, b). We noted that other agents identified in our screen including cilnidipine, dasatinib and venetoclax were also effective in reducing viral entry (fig. S7c, d). Since our pseudovirus also encodes for a GFP reporter, we also assessed this parameter in pseudovirally-infected cells. As noted, both homoharringtonine and halofuginone markedly reduced levels of GFP expression (Fig. 6e), again consistent with their ability to inhibit viral entry. We also tested a separate pseudovirus which encodes for a luciferase-based reporter and showed a similar dose-dependent reduction of the luciferase signal using both homoharringtonine and halofuginone (Fig. S8a, b). In addition, we noted that combined treatment with homoharringtonine and halofuginone was more effective than either agent alone (Fig. S8c).

Finally, given that each permissive cell line has a different reliance on TMPRSS2- versus endosomal-mediated viral entry pathways, we sought to ascertain the effects of our identified compounds on the most clinically relevant cell type, namely primary human respiratory epithelial cells. These primary cells were obtained from normal human lungs under a protocol approved by our Institutional Review Board (IRB) and maintained at an air-liquid interface. In culture, these cells retain features of their normal apical-basal polarity and exhibit the expected bronchial epithelial mucociliary phenotype (31, 32). Similar to what we observed in immortalized cell lines, homoharringtonine and halofuginone were also effective in blocking SARS-CoV-2 pseudoviral entry in these primary human bronchial epithelial cells (Fig. 6f, g). Lastly, we confirmed the role of DCAF1 in regulating the beneficial effects of halofuginone. Of note, DCAF1 knockdown significantly blocked the observed halofuginone-mediated reduction in SARS-CoV-2 pseudoviral entry (Fig. 6h).

## Discussion

By executing an unbiased small molecule screen, we have identified a number of compounds that are currently in active clinical trials or are FDA-approved that can reduce TMPRSS2 expression (Table I). Using the biological IC50 for inhibiting pseudoviral entry, several of these candidates would appear to be clinically viable. The agents identified in our screen may be effective alone, or in combination with each other (Fig. S8c). In addition, as noted initially, SARS-CoV-2 can enter cells through TMPRSS2-mediated pathways, or can employ endo-lysosomal proteases such as cathepsin L to cleave the S protein and gain cell entry (8, 33). If agents such as chloroquine, which modify endosomal pH, are ultimately determined to be effective for COVID-19 infections, we hypothesize that they would likely be synergistic with agents identified here that inhibit TMPRSS2-mediated pathways (fig. S11). Our approach, would also appear to complement strategies that directly target viral replication such as remdesivir (34).

It should be noted that our two most promising leads, homoharringtonine and halofuginone, have been noted to have anti-viral activity (35–37), although the precise mechanism for these effects were not previously determined. Our *in vitro* data would indicate that TMPRSS2 reduction occurs at concentrations that are achievable with the approved dosing in humans (see Table 1). As noted, homoharringtonine is a chemotherapeutic agent that can trigger myelosuppression (22, 23), while halofuginone is generally better tolerated (24, 25). As such, we concentrated our mechanistic studies on the latter, likely more clinically viable agent, demonstrating that TMPRSS2 is targeted for proteasomal-mediated degradation. We have previously described an unbiased siRNA-mediated strategy to rapidly identify which of the more than 600 E3 ubiquitin ligases are relevant for a given target’s half-life (38). Using that platform, we were able to identify DCAF1 as an important regulator of TMPRSS2 stability. Of note, knockdown of DCAF1 raised the basal level of TMPRSS2. More importantly, knockdown of DCAF1 abrogated the ability of halofuginone to trigger a decline in TMPRSS2 levels. It, similarly, reduced the ability of halofuginone to block SARS-CoV2 pseudoviral entry.

These results suggest that halofuginone catalyzes a reduction of TMPRSS2 protein levels through a DCAFl-dependent pathway. This is further supported by the observation that altering a cluster of critical lysine residues on TMPRSS2’s cytoplasmic domain abrogates the effects of halofuginone. Our preliminary data suggests that halofuginone does not inhibit TMPRSS2 catalytic activity (Fig. S9) nor directly binds to either TMPRSS2 or DCAF1 (Fig. S10) suggesting the drug effects may occur indirectly, perhaps by triggering a post-translational modification of TMPRSS2 that favors an enhanced interaction with DCAF1. One such possible post-translational modification comes from previous observations that DCAF1 binding to a substrate can be triggered my mono-methylation of the substrate thereby creating a methyl-degron (39). Whether or not halofuginone can catalyze this or other modifications will require additional study. Of note, halofuginone has also been postulated to act as a glutamyl-prolyl-tRNA synthetase inhibitor, inhibiting translation of a subset of proteins (27). Our data suggests this mechanism may be operative here as well. As such, it appears possible that halofuginone could affect both the translation and the post-translational stability of TMPRSS2.

While we were focusing on TMPRSS2 in this study, recent work has demonstrated that several other host proteases can perform the critical S protein priming step. Notably, SARS-CoV-2 contains an extra “RRAR” amino acid sequence in the S1/S2 domain, making it susceptible to cleavage by the host protease furin during viral packaging (11–14). Furin is a highly expressed proprotein convertase that performs vital cellular functions, and the presence of this furin-like cleavage domain in other viruses is associated with increased pathogenicity and neurotropism (40–42). Future studies are needed to determine whether halofuginone or other promising hits from this study can also reduce furin, or other key host proteases, involved in SARS-CoV2 entry.

Finally, while our results focused on strategies that alter the post-translational stability of TMPRSS2, it should be noted that TMPRSS2 expression is known to be transcriptionally regulated by androgens (43). It is intriguing to speculate whether this may translate into a higher basal level of TMPRSS2 in men, and in turn, whether this higher level of TMPRSS2 expression can partially explain why men appear to be at significantly higher risk for mortality and complications following COVID-19 infection (44). Of note, individuals who have inherited a non-coding SNP that only modestly increases the expression of TMPRSS2, appear to be at a significant elevated risk for developing more severe viral infections (45). As such, we believe that agents identified here, that reduce TMPRSS2 expression, represent a rational approach to modify the clinical course of COVID-19, and potentially future related viral pandemics.

## Supplementary Material

1

## Figures and Tables

**Table 1:** Identified agents’ current clinical indications, human pharmacokinetic properties (46–50) and estimated IC50 for inhibiting viral entry into Caco-2 cells.

Drug	Disease	Regulatory Status	Dose	Route (frequency)	Cmax	In vitro IC50 (viral entry)	Reference
**Homoharringto nine (Omacetaxine)**	Chronic myeloid leukemia (CML)	FDA approved	1.25 mg/m2	SC (BID)	55nM	~30nM	[32]
**Halofuginone**	scleroderma	Phase 1/2	3.5mg/day	Oral	7nM	~30nM	[33]
**Verteporfin**	photosensitizer for photodynamic therapy	FDA approved	0.3mg/kg	IV (within 45min)	1.92μM	~10μM	FDA
**Cilnidipine**	Hypertension	FDA approved	10mg	Oral (QD)	18.1nM	~3μM	[34]
**Dasatinib**	chronic myelogenous leukemia (CML) and acute lymphoblastic leukemia (ALL)	FDA approved	140mg	Oral (QD)	0.307μM	>10μM	[35]
**Venetoclax**	chronic lymphocytic leukemia (CLL) or small lymphocytic lymphoma (SLL)	FDA approved	400mg	Oral (QD)	1.27μM	>10μM	[36]
