# Supplementary material for: A high throughput screen for TMPRSS2 expression identifies FDA-approved and clinically advanced compounds that can limit SARS-CoV-2 entry
Source: Res Sq. 2020 Aug 14:rs.3.rs-48659. Preprint. [Version 1] doi: 10.21203/rs.3.rs-48659/v1 (PMC7430593; doi:10.21203/rs.3.rs-48659/v1)
Supplement: 1 [file NIHPPRS48659V1-supplement-1.pdf]

Supplemental Materials:

MATERIALS AND METHODS

Materials—

High-Capacity cDNA Reverse Transcription Kit (4368814) and SYBR Green PCR Master Mix (4364344) were from Applied Biosystems. BEAS-2B (CRL-9609), Caco-2 (HTB-37), Calu-3 (HTB-55), MLE-12 (CRL-2110) and Vero (CCL-81) cells were all obtained from ATCC. Easy Prep RNA Miniprep Plus Kit (R01-04) was from Bioland Scientific. DC Protein Assay Reagent A/B/S (500-0113/ 0114/ 0115) was from BioRad. DMEM/F-12 (11320082), EMEM (670086), Fetal Bovine Serum (26140079) and Opti-MEM I Reduced Serum Medium (31985062) were from Gibco. Amaxa Nucleofector II was from Lonza. Anti-β-actin antibody (MA5-15739), Countess II Automated Cell Counter (AMQAX1000) and Lipofectamine 3000 Transfection Reagents (L3000015) were from Invitrogen. HIV-1 Gag p24 DuoSet ELISA (DY7360-05) was from R&D Systems. SARS-CoV-2 (2019-nCoV) Spike ORF mammalian expression plasmid (Codon Optimized) (VG40589-UT) was from SinoBiological. The following reagent was obtained through the NIH AIDS Reagent Program, Division of AIDS, NIAID, NIH: HIV-1 pNL4-3 ΔEnv Vpr Luciferase Reporter Vector (pNL4-3.Luc.R-E-) from Dr. Nathaniel Landau. pSF-CMV-VSVG (OG592) VSV G Expression Plasmid (SnapFast Pro) (OG592) was from Oxgene. AsiSI (R0630), Eco53kI (R0116S), PmeI (R0560), Quick Ligation Kit (M2200) were from New England Biolabs (NEB). Mouse monoclonal anti–V5 Tag (R960-25) and TMPRSS2 antibody (PA5-83286) were from Thermo Fisher. X-tremeGENE HP DNA Transfection Reagent (6366244001) and X-tremeGENE siRNA Transfection Reagent (4476115001) were from Sigma-Aldrich. Lenti-X GoStix Plus (631280) were from Takara. Venetoclax (HY-15531) and Homoharringtonine (HY-14944) were from MedChemExpress. Halofuginone (S8144) and Cilnidipine (S1293) were from

SelleckChem. Bafilomycin A1 (11038), Carfilzomib (17554), Dasatinib (11498), and Verteporfin (17334) were from Cayman Chemical. Antibody against E-cadherin (G-10, sc-8426) was from Santa Cruz Biotechnologies. MG132 (F1100) was from UBPBio. CellTiter-Glo 2.0 Cell Viability Assay (G9243), Nano-Glo HiBiT Lytic Detection System (N3040), Nano-Glo HiBiT Extracellular Detection System (N2421), Nano-Glo HiBiT Blotting System (N2410), HiBiT CMV-neo Flexi Vectors (N2401, N2391) were from Promega.

Cell Culture— Beas-2b and MLE-12 cells from ATCC were cultured in HITES media supplemented with 10% fetal bovine serum (FBS). Caco-2 and HEK293T were from ATCC and cultured in DMEM (Gibco) supplemented with 10% FBS. Calu-3 and Vero cells were from ATCC and cultured in EMEM (ATCC) supplemented with 15% FBS. For the generation of primary bronchial epithelial cells, following attaining informed consent, airway segments and lung tissue were obtained from excess pathological tissue following lung transplantation in accordance with a protocol approved by the University of Pittsburgh Investigational Review Board (*32*). The isolation, growth and maintenance of these cells were as previously described (*31, 32*). Cells were treated with compound at indicated doses for indicated times, or at the following doses: HFG, 3μM; Carfilzomib, 1μM; Bafilomycin A1, 1μM.

Cloning— HiBiT-tagged TMPRSS2 and SARS-2-CoV spike protein plasmid constructs were generated using molecular cloning and the FLEXI system (Promega). Briefly, the open reading frame of the target genes were PCR amplified with restriction sites for AsiSI and PmeI and was cloned into pFC37K-HiBiT plasmid. Point mutants (Lysine→Arginine) were generated through QuikChange II XL Site-Directed Mutagenesis Kit (Aglient). All plasmid constructs were verified by DNA sequencing (Genewiz).

Transfection— Plasmid transfections were conducted using nucleofection in Beas-2b and MLE-12 cells using Nucleofector II (Amaxa). X-tremeGENE HP DNA transfection reagent or Lipofectamine 3000 transfection reagent was used for plasmid transfections of HEK293T cells.

FDA-Approved Compound Library Screening— Human bronchial epithelial Beas-2b cells transiently expressing TMPRSS2-HiBiT (C-terminal) were seeded to a final density of 1×10^4 cells per well. The FDA-approved compound library (100nL per drug) was stamped to 384-well tissue culture plates using CyBio Well vario (Analytik Jena). Compounds were plated to the final concentrations of 10 μM. After 18hr of treatment, culture media was removed and cells were processed for Nanoluciferase activity using Extracellular HiBiT detection system (Promega), according to manufacturer's protocol. After reading the extracellular HiBiT signals, TritonX-100 solution was added into each well (final concentration 0.05%) for cell lysis, and HiBiT signals were acquired again for all plates. Signals were collected and quantified using a Cytation 5 plate reader from Biotek. For secondary screening to determine drug IC50, specific compounds were cherry-picked using a TTP Mosquito X1 followed by serial dilutions of compounds were prepared using a Bravo automated liquid handling platform (Agilent). Cell seeding and extracellular and lytic HiBiT signal acquisition were performed according to the same protocol described above.

High-Throughput Liquid Handling— A Thermo Scientific custom HTS platform and Agilent Bravo automated liquid-handling platform was used to transfer contents of a FDA-approved compound library into assay plates. Biotek EL406 washer dispenser was used to distribute reagents or cell solutions into assay plates. For multiple plates operation, plate and liquid handling sequence and intervals were controlled through the Agilent VWORKs software.

Cell Viability Assessment— Cell viability was tested using CellTiter-Glo 2.0 Cell Viability Assay (Promega). 20µl reagent was dispensed directly into each well of the 384-well tissue culture plates prior to luminescence signal acquisition by Cytation 5 plate reader.

Compound Washout Assays— Cells were pre-treated with indicated compound for 18-hr prior to 1 round of washing and incubation with fresh culture media for the indicated time periods. After the indicated times, cells were collected and processed for TMPRSS2 immunoblotting.

RT-qPCR — Total RNA was extracted using RNA Extraction Miniprep Kit from Bioland Scientific, following the manufacturer's protocol. cDNA was prepared using High-Capacity RNA-to-cDNA Kit from Applied Biosystems. SYBR Green Real-Time PCR Master Mixes from Applied Biosystems were used in qPCR, detecting the expression level TMPRSS2, HPN, ST14 and CORIN.

Ubiquitination Assay — TMPRSS2-V5-HIS in pcDNA3.1D was co-expressed with HA-DCAF1 in pcDNA3 in Beas-2B cells for 18hr, prior to lysis and precipitation with Dynabead HIS-resin (Thermo). Precipitate was eluted in 1xLaemmli Protein Sample Buffer at 95C for 10min, and resolved through SDS-PAGE immunoblotting.

Ubiquitination siRNA screen — TMPRSS2-HiBiT cells were screened with an siRNA library targeting Ubiquitination-related machinery as previously described (*38*). Briefly, 25 ng of siRNA was mixed with Lullaby transfection reagent (OzBiosciences) and diluted in Opti-MEM media. The transfection mixture incubated at room temperature for 20 minutes, and was added to 20µL of HITES+10%FBS media with 2000 cells. Following 72hr knockdown, Lytic HiBiT luciferase assays were performed using manufacturer’s protocol. For conformational specific gene silencing, small interfering RNAs were selected and purchased from IDT, and transfected in cells using

Lullaby siRNA transfection reagent, with Negative Control DsiRNA transfected as control. Subsequent analysis was performed after 72 h of knockdown.

In vitro transcription and translation — TMPRSS2 constructs were synthesized in vitro using TnT Coupled Reticulocyte Lysate System (Promega) following manufacturer's protocol. HFG plus proline assays were conducted as previously described (*27*), with free amino acids diluted 5-fold and proline supplemented at indicated concentrations.

Immunoblotting—Cells were lysed in RIPA buffer supplemented with EDTA-free protease inhibitor tablet on ice. Cell lysates were sonicated at 20% amplification for 12 seconds and centrifuged at 12,000 g for 10 minutes at 4°C. Supernatants were collected and normalized for the total protein concentrations, mixed with 6X protein sample buffer, and incubated at 42°C for 10 minutes. Sample lysate was resolved using 4-20% acrylamide PROTEAN® TGX™ precast gels from BioRad and electrophoresed in TGS buffer. The proteins were then electro-transferred to nitrocellulose membranes. Blots were incubated in 15 ml of blocking buffer for 1 hr at room temperature, before incubation in 10 ml of the primary antibody solution (1:2000 dilution) overnight at 4°C. Afterwards, three 10-minute washing were performed in 15ml TBST. Blots were then incubated with 10 ml of the secondary antibody solution for 1 hr at room temperature. After three 10-minute washing in 15ml TBST, blots were then developed using West Femto Maximum Sensitivity Substrate from Thermo Scientific, and imaged using ChemiDoc Imaging System from Bio-Rad.

HiBiT Blotting— Samples from cells transfected with HiBiT-tagged proteins were prepared following the same protocol as immunoblotting. Proteins were transferred to a nitrocellulose membrane, followed by gentle rocking in TBST to rinse away transfer buffer. Nano-Glo HiBiT blotting system (Promega) was used for development, following manufacturer's protocol. Briefly,

the blot was incubated in 5 ml 1× Nano-Glo blotting buffer supplemented with 25 µl LgBiT protein overnight at 4°C. The next day, 10 µl Nano-Glo luciferase assay substrate was directly added into the solution and mixed well immediately. After incubation for 5 min at room temperature in dark, the blot was imaged by ChemiDoc Imaging System (Bio-Rad), using chemiluminescence mode.

Pseudovirus Entry Assays— Pseudovirus with SARS-CoV-2 spike protein with a C-terminal HiBiT tag was generated by co-transfection of 293T cells with psPAX2 (Addgene, MA), pLenti-c-mGFP (Origene, MD) and PFC37K-HIBIT-SARS-CoV-2-S (Backbone was from Promega, WI) by using lipofectamine 3000 (Invitrogen, CA). Briefly, 293T cells were seeded one day before in 8 ml DMEM complete media without antibiotics in a 10 cm tissue culture dish. The following morning, cells were transfected with 8 µg psPAX2, 8 µg pLenti-c-mGFP or pLenti-c-FLUC and 4 µg PFC37K-HIBIT-SARS-CoV-2-S with lipofectamine 3000 according to the manufacturer's protocol. Six hours later, media was changed with fresh full media without antibiotics. The supernatants were harvested 48h post-transfection, and centrifuged at 500 × g for 10 min to remove cell debris. Virus titer was checked by Lenti-X GoStix Plus (Takara,CA). HibiT expression was checked by Nano-Glo® HiBiT Lytic Detection System (Promega, WI). The HIV-1 Gag p24 content in the produced SARS-CoV-2-HiBiT pseudovirus was quantified by ELISA (R&D systems), following the manufacturer's protocol. To detect infectivity, various cell types with control or compound treatment were incubated for 1h with pseudovirus, after extensive washes with PBS, cells were lysed with Nano-Glo HiBiT lytic reagent (Promega) for 20 min. Luminescence signals were then acquired by ClarioSTAR microplate reader (BMG Labtech, Cary, NC).

Immunocytochemistry— Caco-2 cells were seeded in 384-well glass-bottom plates (Cellvis, 5000 cells/well) and treated with halofuginone or homoharringtonine at the indicated concentrations for

18hr. Cells were then treated with a pseudovirus encoding the SARS-CoV-2 Spike protein and mGFP. Forty-eight hours after infection, cells were fixed (4% paraformaldehyde), permeabilized (0.5% Triton X-100), and stained for GFP (Cell Signaling Technology), followed with a goat anti-rabbit Alexa Fluor 568 secondary antibody. The fluorescent signal was imaged using Image Express (Molecular Devices) to measure viral entry.

TMPRSS2 activity assay— Test compounds were pre-incubated with recombinant TMPRSS2 (AA 106-492, LSBio) in assay buffer (TBS, 0.05% Tween-20) for 15 minutes before addition of Boc-Gln-Ala-Arg-MCA (Peptides International) substrate to a final concentration of 10 µM. Final concentration of TMPRSS2 was 1.35 µg/mL. Fluorescence (380-15nm excitation/470-20nm emission) of the samples was monitored over 4:45 hours and the slope of the curves was calculated by linear regression analysis and used as output for TMPRSS2 activity.

Cellular thermal shift assay— B2B cells were transfected with TMPRSS2-HiBiT plasmid overnight, prior to treatment with vehicle or HFG (3µM for 1 hr). Cells were collected and resuspended in 10 ml of PBS supplemented with EDTA-free protease inhibitor tablet. Cell solutions were aliquoted to 10 PCR microtubes evenly. Using the PCR thermocycler generating temperature gradient, each aliquot was incubated at a certain temperature between 40~58°C with 2°C interval for 3 min, then at room temperature for 3 min. Samples were immediately snap frozen in liquid nitrogen and 2 cycles of freeze-thaw were followed. After vortex briefly, samples were transferred to 1.7 ml microcentrifuge tubes for centrifuging at 20,000 g, 4°C for 20 min. Supernatants were carefully acquired and used for subsequent immunoblotting analysis. The whole procedure followed the protocol described in previous literature (*51*).

Statistics — Statistical comparisons were performed in GraphPad Prism. Unpaired two-tailed Student's *t*-test was used to compare two groups. Comparisons of more than two groups were tested with one-way ANOVA with Tukey's post-hoc test of multiple comparisons.

Supplemental Figure Legends:

Supplementary Figure 1: Assessment of TMPRSS2 HTS Screen. TMPRSS2-HiBiT expressing BEAS-2B cells were seeded into 384-well plates for 24h. Half of the cells were treated with CHX overnight and the lytic HiBiT signal was measured. A subsequent Z'-factor was calculated.

Supplementary Figure 2: IC50 calculations for compounds that reduce TMPRSS2 expression. Remaining compounds (top 24 in total) inhibiting TMPRSS2 expressions were tested for their potency in reducing extracellular (first column) or total (middle column) TMPRSS2-HiBiT protein levels. An assessment of cellular toxicity for each compound (CellTiter-Glo) is shown in the last column.

Supplementary Figure 3: Agents identified in the screen selectively decrease TMPRSS2 in airway cells. TMPRSS2-HiBiT expressing MLE-12 cells were treated with three agents identified in the screen (homoharringtonine, halofuginione, or venetoclax) at the indicated concentration for 18 hr prior to chemiluminescent detection of TMPRSS2-HiBiT expression. Conventional detection of actin is shown as a loading control.

Supplementary Figure 4: Analysis of TMPRSS2 mRNA expression by qPCR analysis of Caco-2 cells treated with homoharringtonine or halofuginone for 6 hr. Expression of other related proteases including hepsin (HPN), ST14, and corin were also measured. Data represent mean -/+ SEM (n = 4). *$P < 0.05$, relative to vehicle, or as indicated by one-way ANOVA with Dunnett's test of multiple comparisons.

Supplementary Figure 5: (**A-B**) Calu-3 cells were treated with a dose course of homoharringtonine (HHT) or halofuginone (HFG) for 18 hr prior to immunoblotting analysis. Densitometry of endogenous TMPRSS2 is shown below. Data represent mean -/+ SEM (n=3). (**C**) Time-course treatment of HHT-treated Caco-2 cells (3 µM). TMPRSS2 densitometry is shown, data are mean +/- SEM (n=3). (**D**) Immunoblot analysis of Caco-2 cells treated with HHT for 18hr prior to removing the drug, adding fresh media, and then analyzing the protein recovery time course.

Supplementary Figure 6: (**A)** in vitro transcription/translation assay. V5- or HiBiT-tagged TMPRSS2 was treated with HFG without or with L-proline supplementation (n=3). **(B)** HFG-induced decrease in TMPRSS-HiBiT signal is blunted with exogenous proline addition to Beas-2B cells. Data represent mean +/- SEM (n=8).

Supplementary Figure 7: (**A-B**) SARS-CoV-2 pseudovirus entry assay in Calu-3 cells following HHT or HFG treatment, corrected for cell viability with Celltiter-Glo and normalized to vehicle treatment. (**C**) SARS-CoV-2 pseudovirus entry assay in Caco-2 cells treated with calcium channel blocker cilnidipine. (**D**) Treatment of Caco-2 cells with the indicated agents. All agents were assessed at 10 µM except for camostat (3 µM). data are mean -/+ SEM n=3, SARS-CoV-2 pseudoviral luminescence is corrected for cell viability with CellTiter-Glo and normalized to vehicle treatment. *P $<$0.05, relative to 0 time point or control, or as indicated by one-way ANOVA with Dunnett's test of multiple comparisons (A-B).

Supplementary Figure 8: (A-B) Effects of HHT or HFG on pseudoviral-mediated FLUC expression in Caco2 cells. data are mean -/+ SEM (n=4)., SARS-CoV-2 pseudoviral FLUC is corrected for cellular protein concentration and normalized to vehicle treatment. (**C**) Pseudoviral entry assay with HHT (10 nM), HRG (10 nM) alone or combined, data are mean -/+ SEM (n=3). *P $<$0.05, relative to 0 time point or control, or as indicated by one-way ANOVA with Dunnett's test of multiple comparisons (A-C).

Supplementary Figure 09: in vitro fluorescent activity assay of recombinant TMPRSS2 in the presence of a dose course of several compounds (N=4)

Supplementary Figure 10: Cellular thermal shift assay of TMPRSS2 (**A**) and DCAF1 (**B**) proteins in B2B cells transfected with TMPRSS2-HiBiT plasmid, upon treatment with vehicle or HFG. The abundance of TMPRSS2 and DCAF1 were detected by HiBiT blotting and anti-DCAF1 antibody respectively. The corresponding quantifications of densitometry were shown beneath.

Supplementary Figure 11: Strategies to limit SARS-CoV-2 infection. Agents identified here act by reducing the expression of TMPRSS2 and thereby limiting viral entry. Combinations of these agents may synergize which each other to reduce TMPRSS2 levels below what a single agent can achieve. In addition, agents that reduce TMPRSS2 levels or function would also likely synergize with agents (e.g. chloroquine) that predominantly target viral entry through the endosomal pathway.

Fig. S1

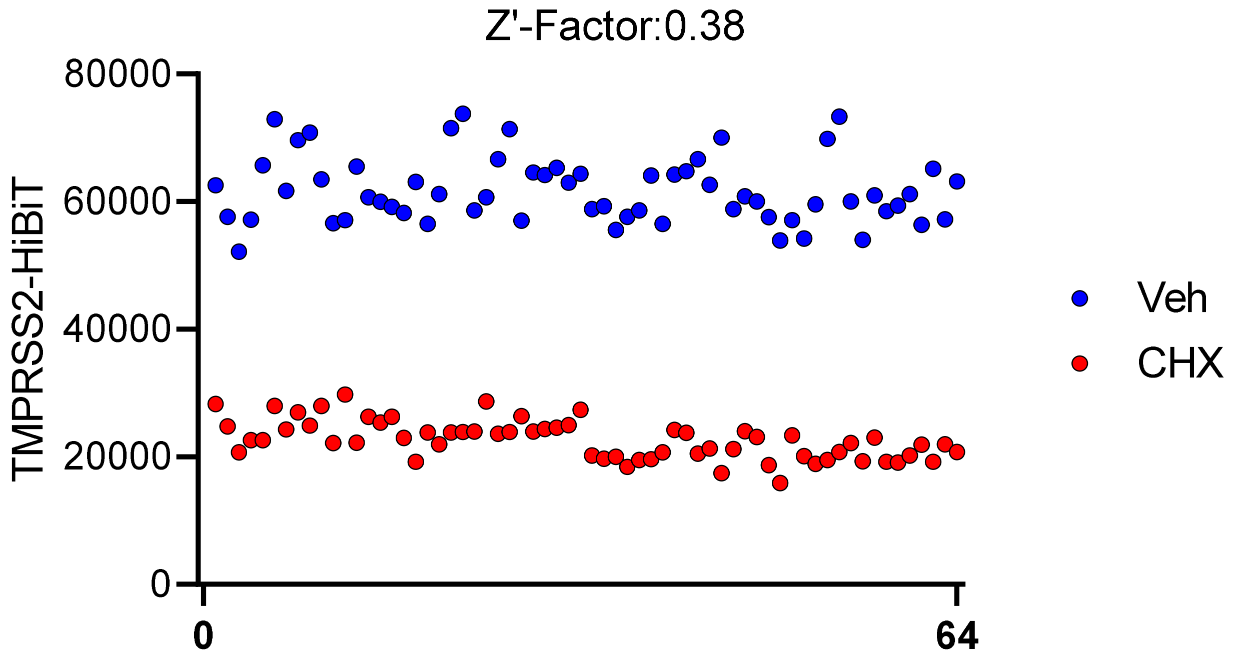
Z'-Factor:0.38
80000
60000
40000
20000
0
TMPRSS2-HiBiT
0
64
Veh
CHX

Fig. S2

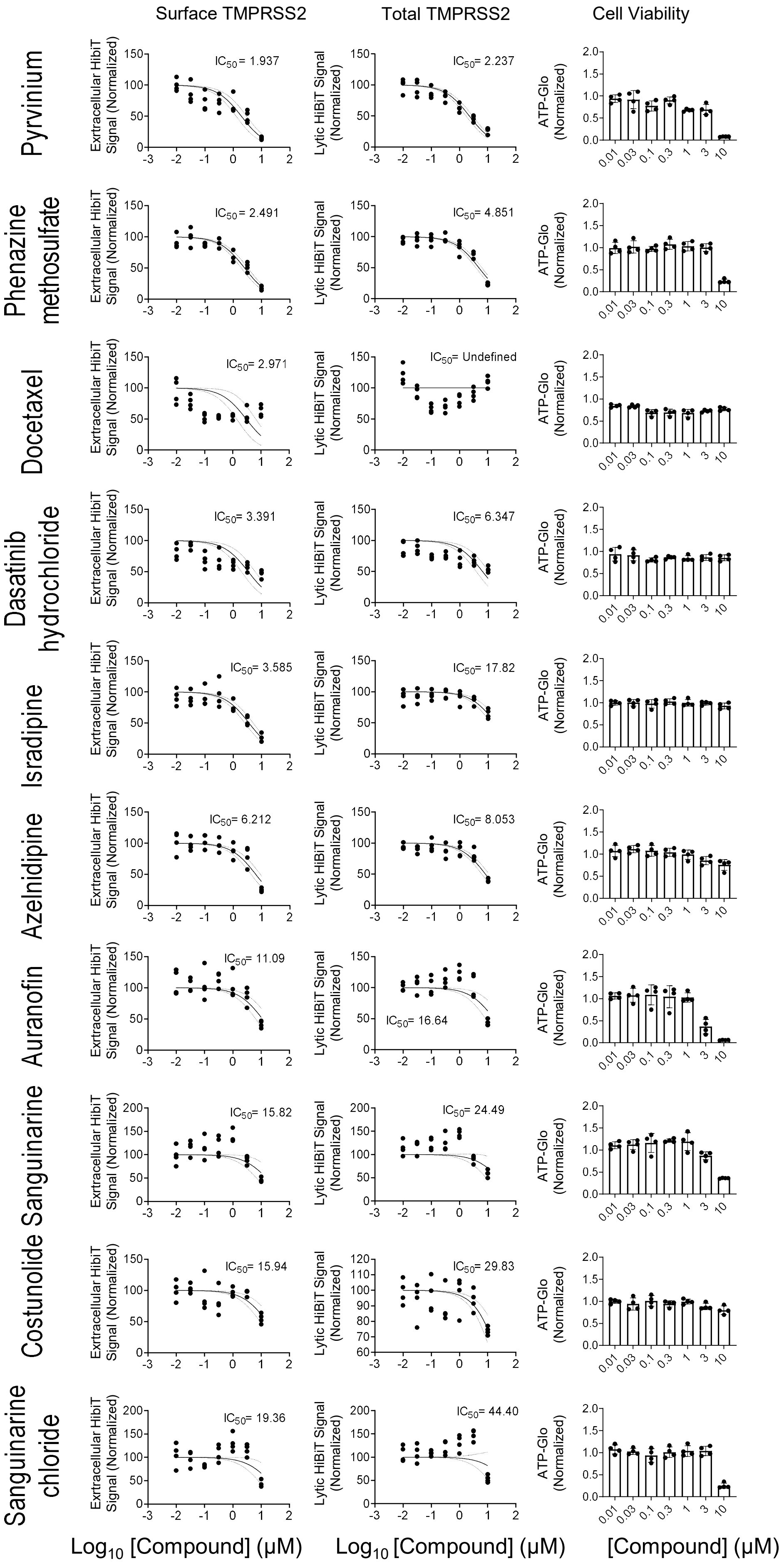
Surface TMPRSS2
Total TMPRSS2
Cell Viability
Pyrvinium
IC50= 1.937
IC50= 2.237
Phenazine methosulfate
IC50= 2.491
IC50= 4.851
Docetaxel
IC50= 2.971
IC50= Undefined
Dasatinib hydrochloride
IC50= 3.391
IC50= 6.347
Isradipine
IC50= 3.585
IC50= 17.82
Azelnidipine
IC50= 6.212
IC50= 8.053
Auranofin
IC50= 11.09
IC50= 16.64
Sanguinarine
IC50= 15.82
IC50= 24.49
Costunolide
IC50= 15.94
IC50= 29.83
Sanguinarine chloride
IC50= 19.36
IC50= 44.40
Exrtracellular HibiT Signal (Normalized)
Extracellular HibiT Signal (Normalized)
Lytic HiBiT Signal (Normalized)
ATP-Glo (Normalized)
Log10 [Compound] (μM)
Log10 [Compound] (μM)
[Compound] (μM)

Fig. S3

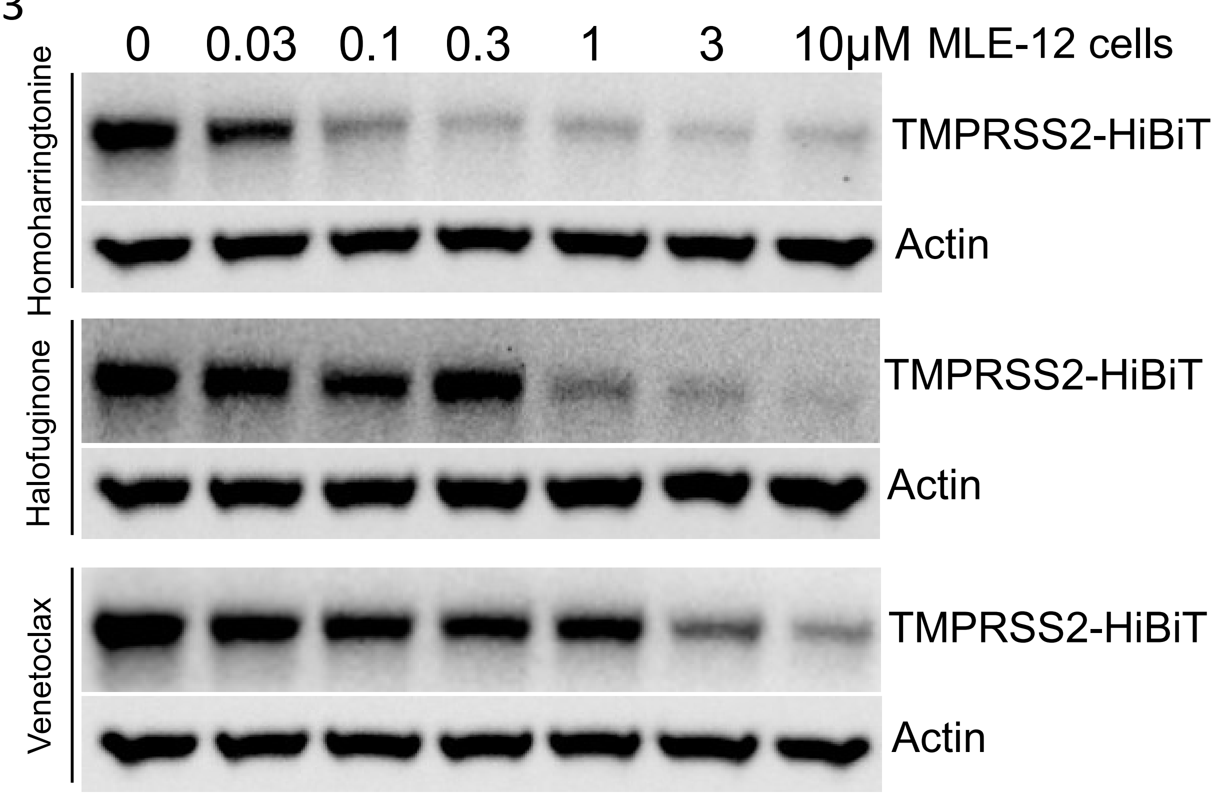

0
0.03
0.1
0.3
1
3
10μM
MLE-12 cells
Homoharringtonine
TMPRSS2-HiBiT
Actin
Halofuginone
TMPRSS2-HiBiT
Actin
Venetoclax
TMPRSS2-HiBiT
Actin

Fig. S4

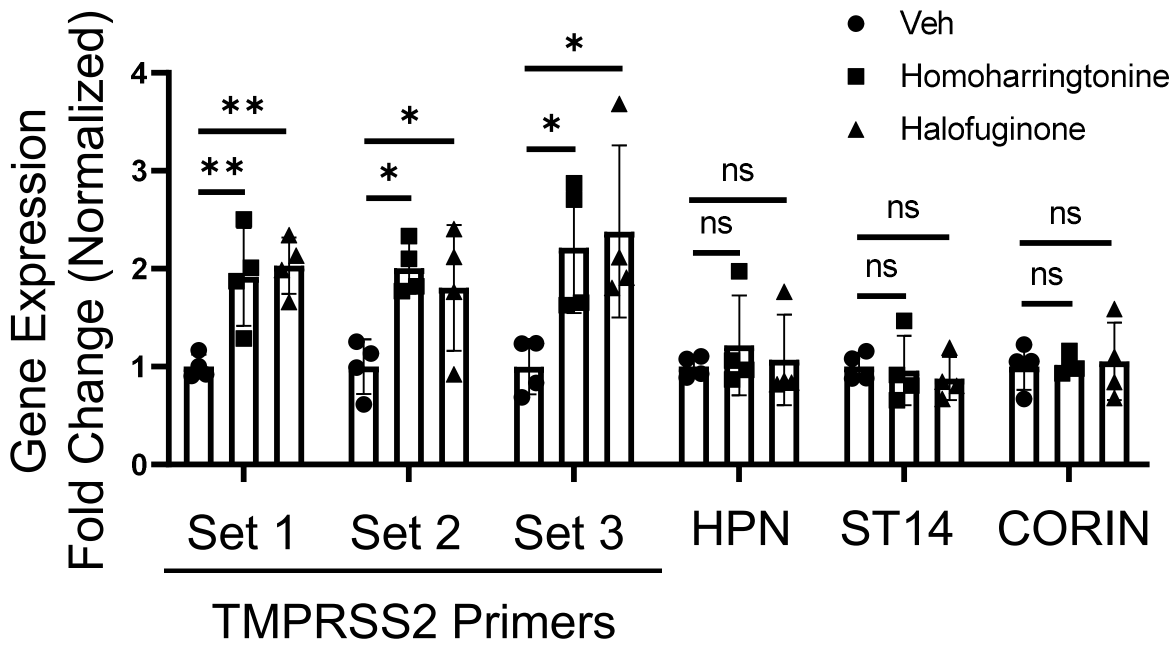
Veh
Homoharringtonine
Halofuginone
Gene Expression
Fold Change (Normalized)
0
1
2
3
4
**
**
*
*
*
*
ns
ns
ns
ns
ns
ns
Set 1
Set 2
Set 3
HPN
ST14
CORIN
TMPRSS2 Primers

Fig. S5

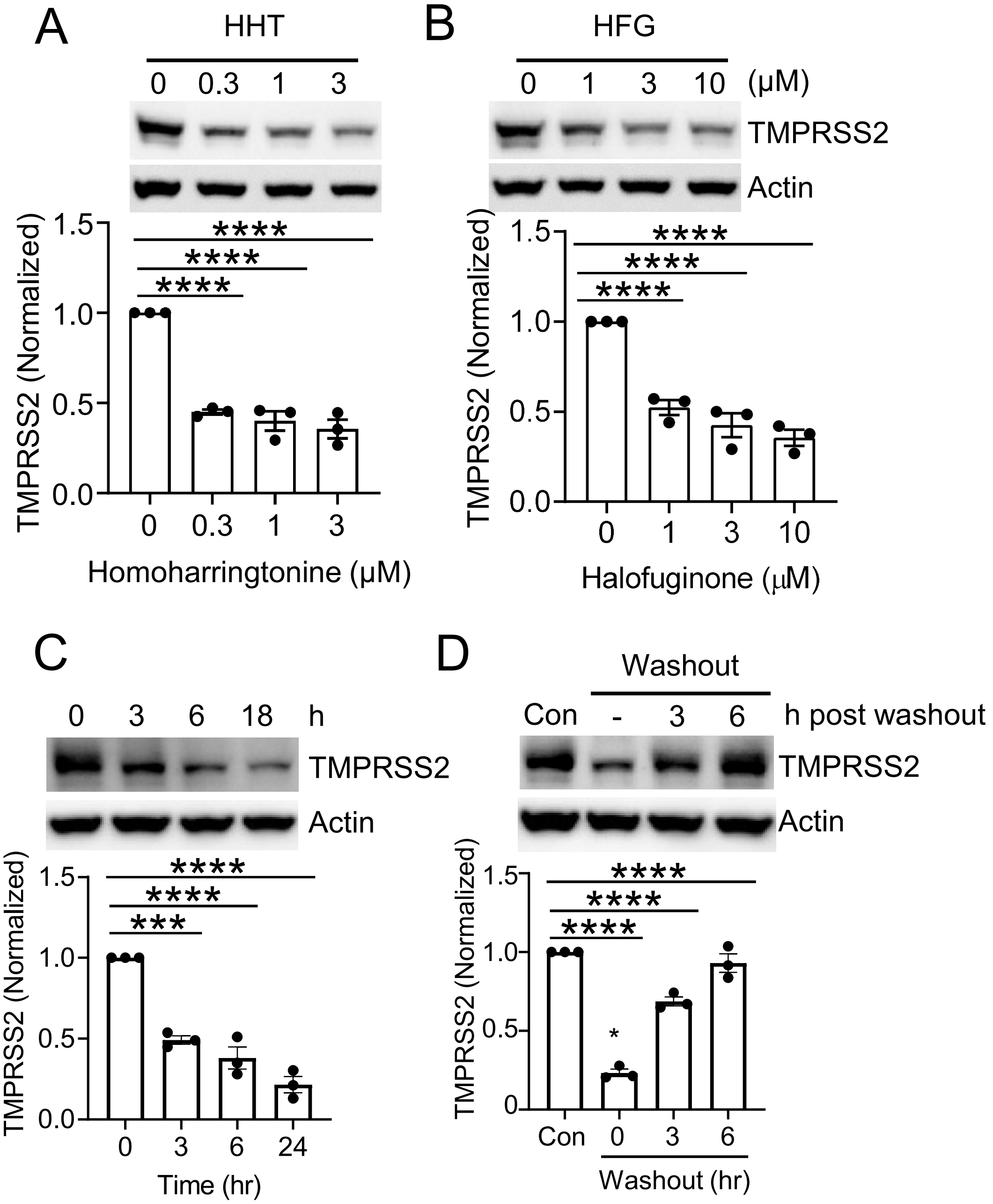
A
HHT
0 0.3 1 3
TMPRSS2 (Normalized)
1.5
1.0
0.5
0.0
****
****
****
0 0.3 1 3
Homoharringtonine (µM)
B
HFG
0 1 3 10 (µM)
TMPRSS2
Actin
TMPRSS2 (Normalized)
1.5
1.0
0.5
0.0
****
****
****
0 1 3 10
Halofuginone (µM)
C
0 3 6 18 h
TMPRSS2
Actin
TMPRSS2 (Normalized)
1.5
1.0
0.5
0.0
****
****
***
0 3 6 24
Time (hr)
D
Washout
Con - 3 6 h post washout
TMPRSS2
Actin
TMPRSS2 (Normalized)
1.5
1.0
0.5
0
****
****
****
*
Con 0 3 6
Washout (hr)

Fig. S6

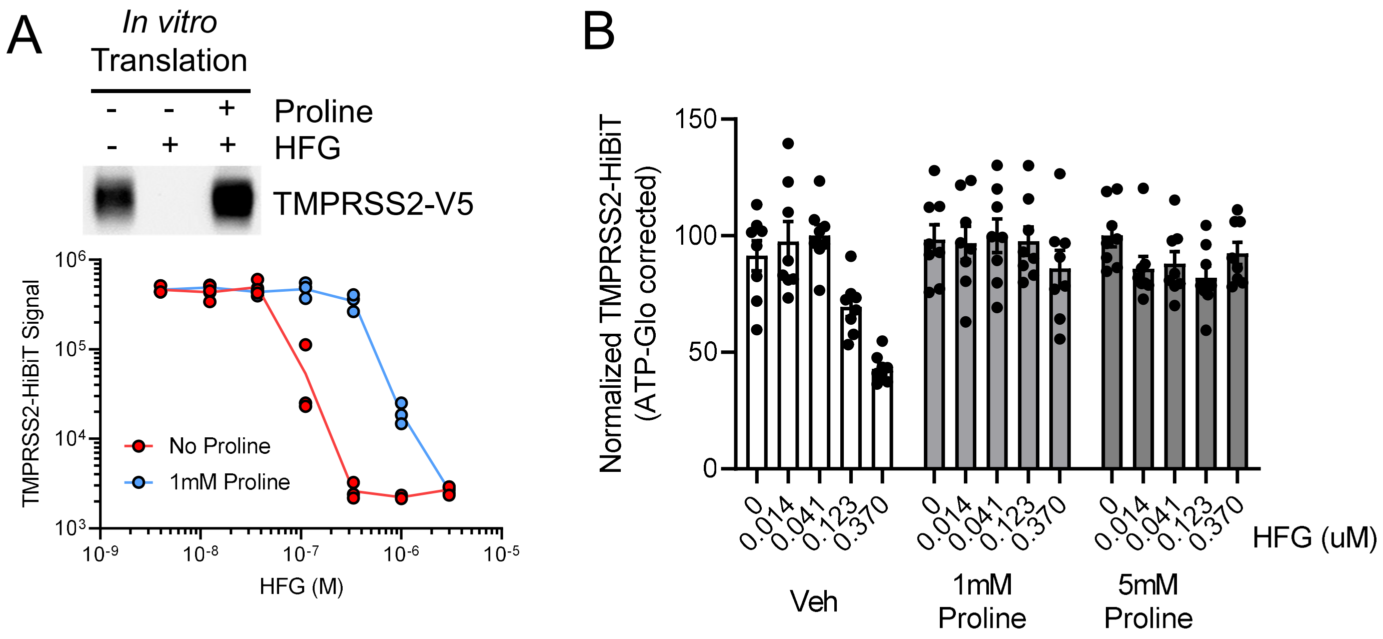
A
In vitro Translation
Proline
HFG
TMPRSS2-V5
TMPRSS2-HiBiT Signal
No Proline
1mM Proline
HFG (M)
B
Normalized TMPRSS2-HiBiT (ATP-Glo corrected)
0 0.014 0.041 0.123 0.370
HFG (uM)
Veh
1mM Proline
5mM Proline

Fig. S7

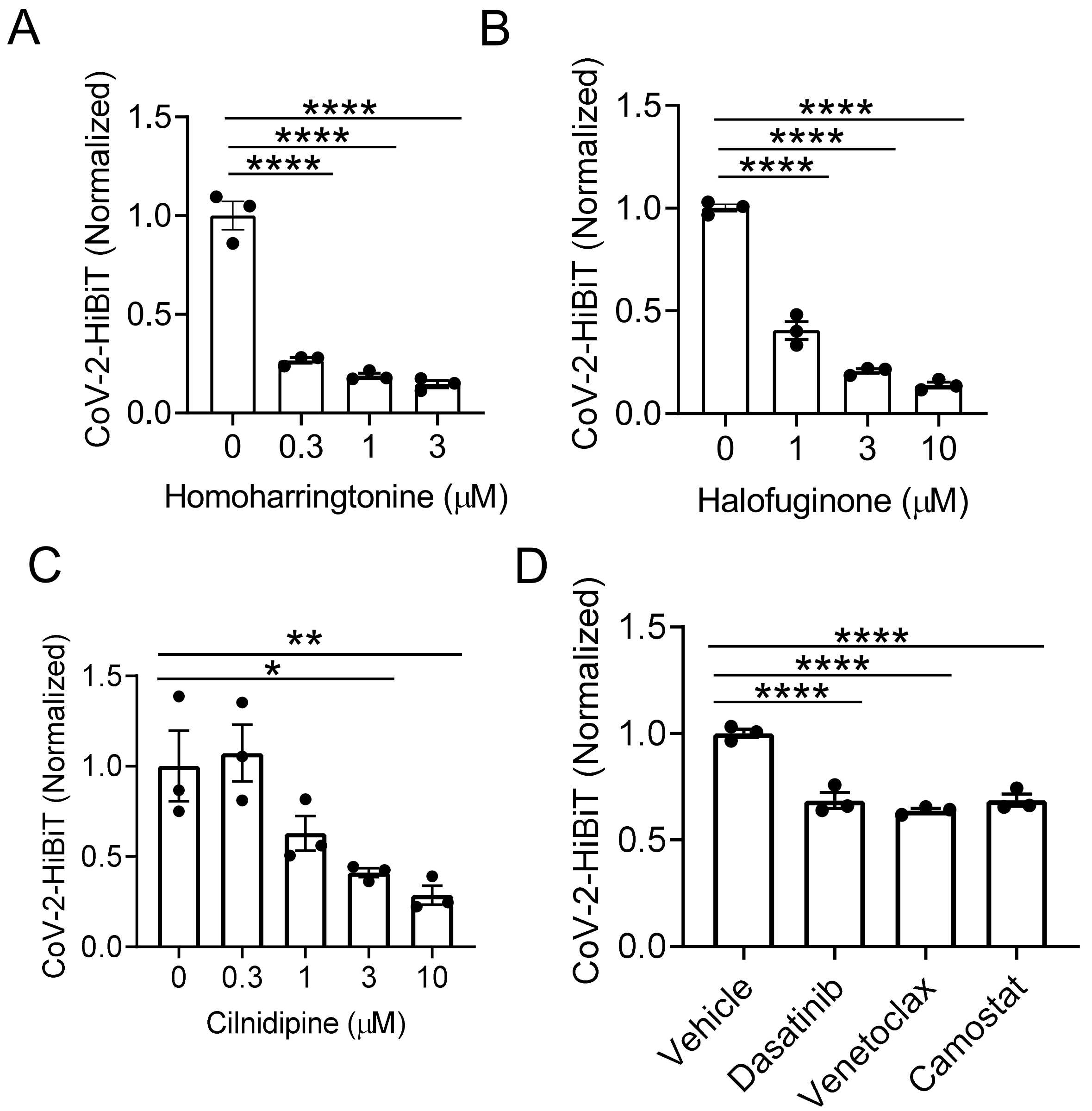
A
CoV-2-HiBiT (Normalized)
1.5
1.0
0.5
0.0
****
****
****
0
0.3
1
3
Homoharringtonine (μM)
B
CoV-2-HiBiT (Normalized)
1.5
1.0
0.5
0.0
****
****
****
0
1
3
10
Halofuginone (μM)
C
CoV-2-HiBiT (Normalized)
1.5
1.0
0.5
0.0
**
*
0
0.3
1
3
10
Cilnidipine (μM)
D
CoV-2-HiBiT (Normalized)
1.5
1.0
0.5
0.0
****
****
****
Vehicle
Dasatinib
Venetoclax
Camostat

Fig. S8

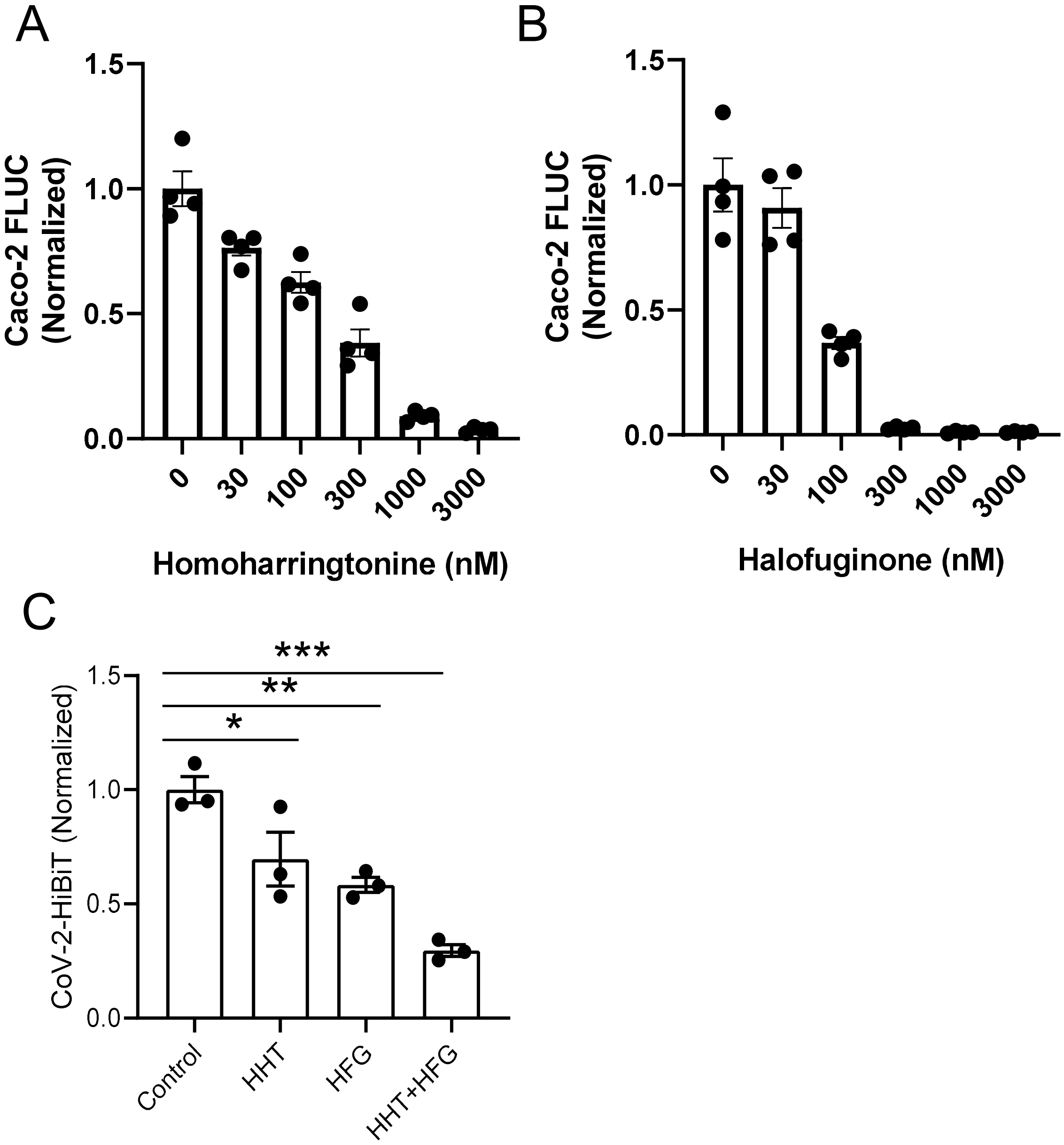

A
B
C
Caco-2 FLUC (Normalized)
1.5
1.0
0.5
0.0
0
30
100
300
1000
3000
Homoharringtonine (nM)
Halofuginone (nM)
***
**
*
CoV-2-HiBiT (Normalized)
Control
HHT
HFG
HHT+HFG

Fig. S9

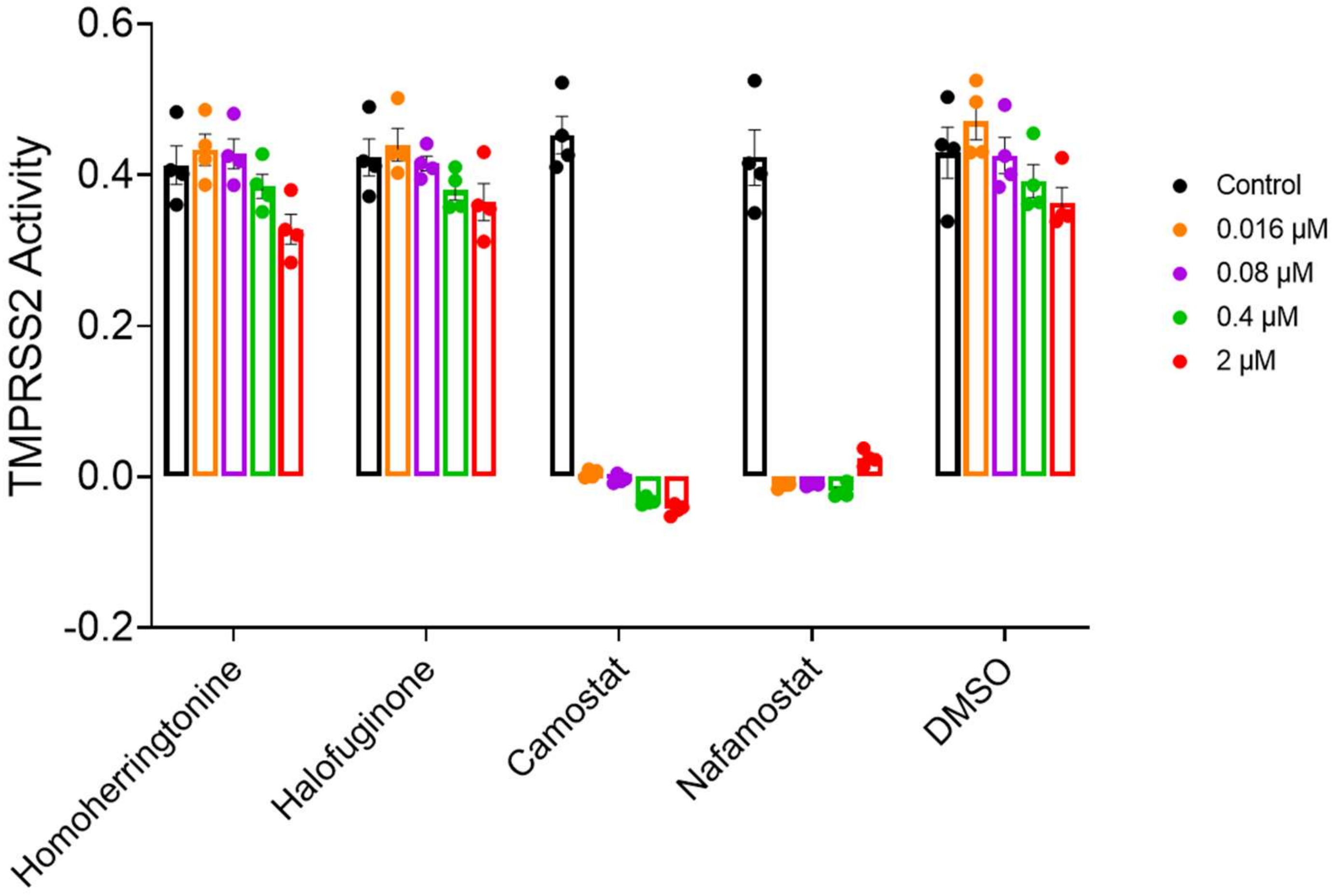

0.6
0.4
0.2
0.0
-0.2
TMPRSS2 Activity
Homoherringtonine
Halofuginone
Camostat
Nafamostat
DMSO
Control
0.016 µM
0.08 µM
0.4 µM
2 µM

Fig. S10

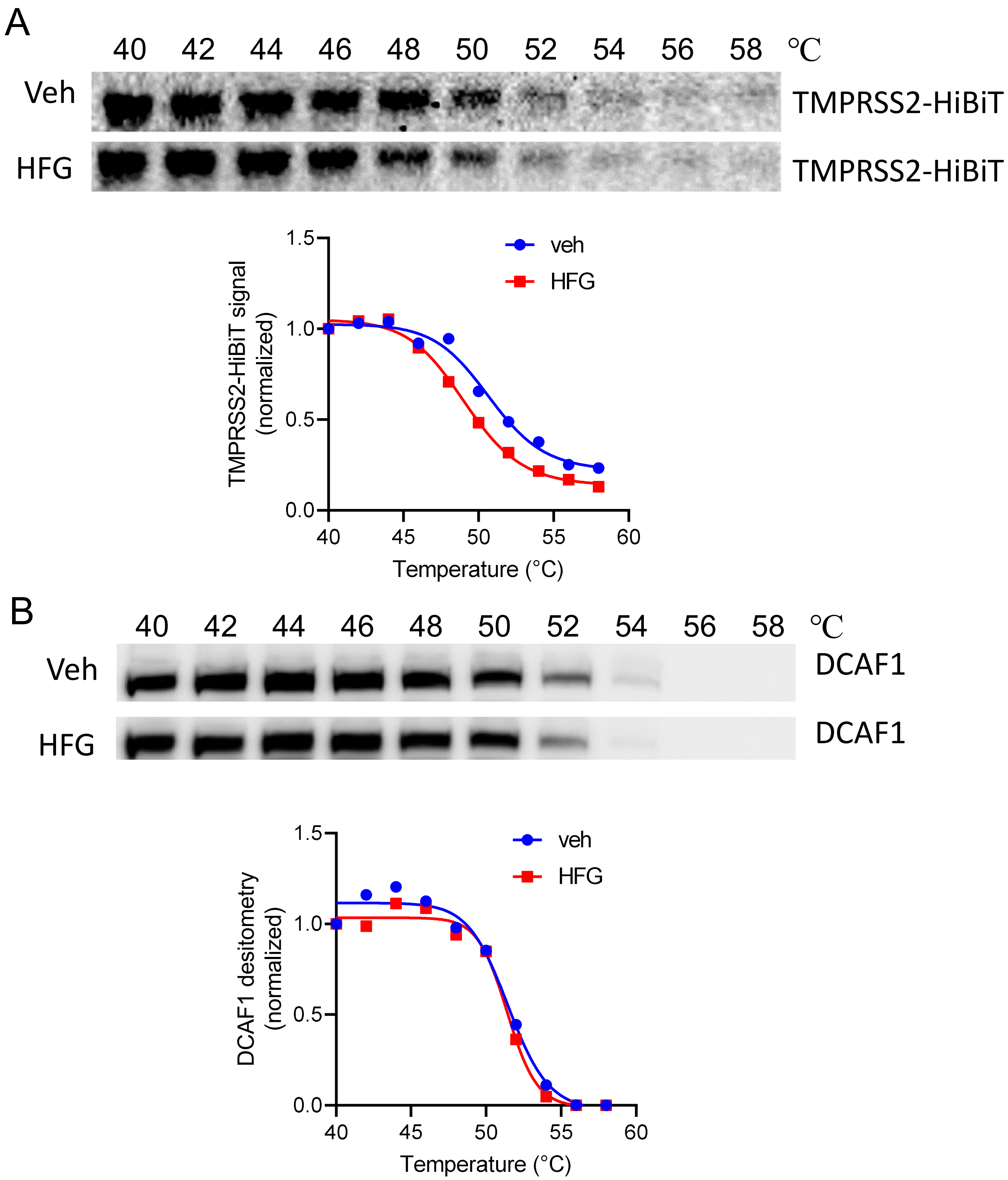
A
40
42
44
46
48
50
52
54
56
58
°C
Veh
TMPRSS2-HiBiT
HFG
TMPRSS2-HiBiT
TMPRSS2-HiBiT signal (normalized)
1.5
1.0
0.5
0.0
40
45
50
55
60
Temperature (°C)
veh
HFG
B
40
42
44
46
48
50
52
54
56
58
°C
Veh
DCAF1
HFG
DCAF1
DCAF1 desitometry (normalized)
1.5
1.0
0.5
0.0
40
45
50
55
60
Temperature (°C)
veh
HFG

Fig. S11

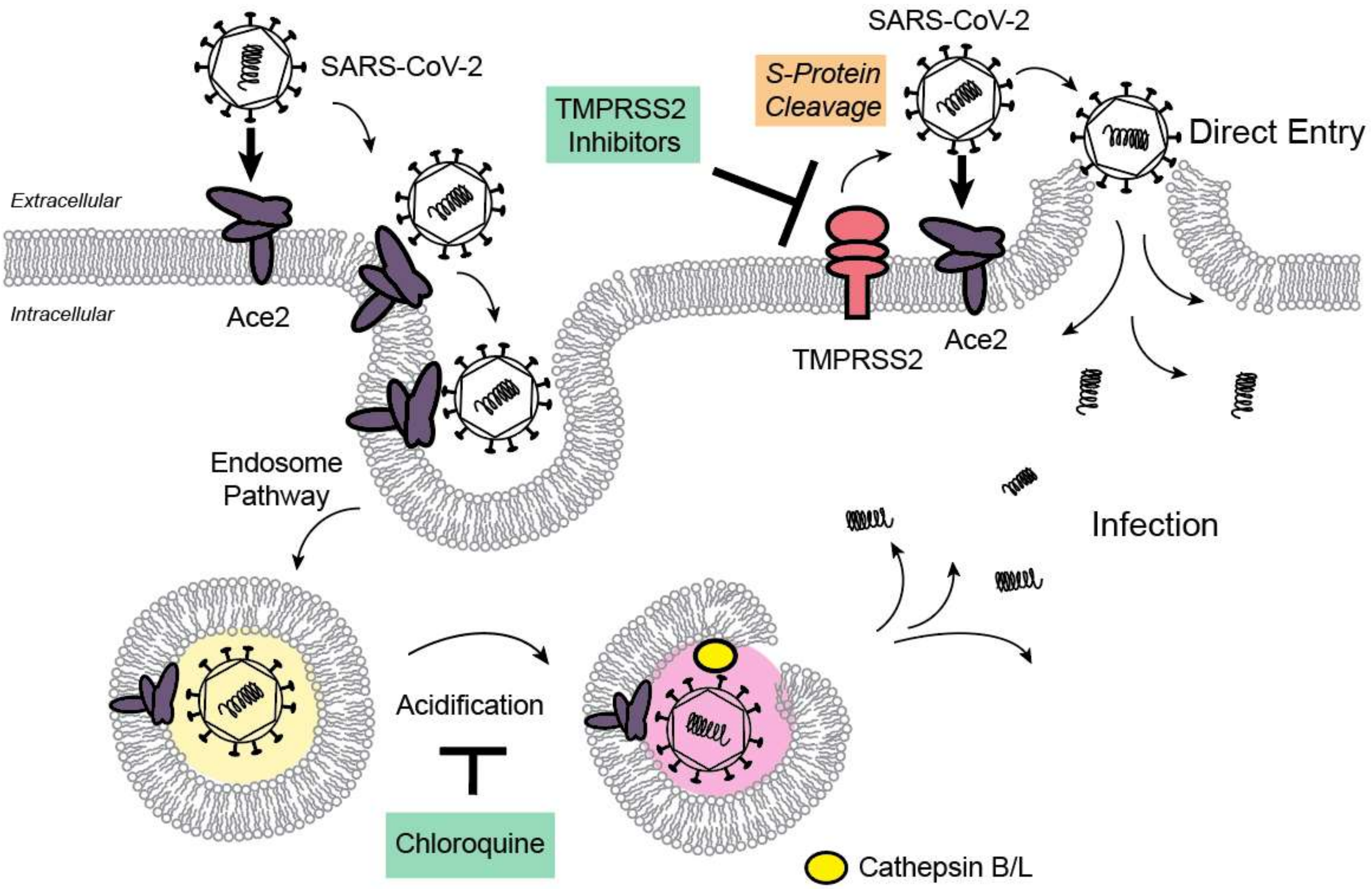
SARS-CoV-2
Extracellular
Intracellular
Ace2
Endosome
Pathway
TMPRSS2
Inhibitors
S-Protein
Cleavage
SARS-CoV-2
Direct Entry
TMPRSS2
Ace2
Infection
Acidification
Chloroquine
Cathepsin B/L
